# Clinical practice guidelines for the diagnosis and treatment of adult diffuse glioma‐related epilepsy

**DOI:** 10.1002/cam4.2362

**Published:** 2019-06-26

**Authors:** Shuli Liang, Xing Fan, Ming Zhao, Xia Shan, Wenling Li, Ping Ding, Gan You, Zhen Hong, Xuejun Yang, Guoming Luan, Wenbin Ma, Hui Yang, Yongpin You, Tianming Yang, Liang Li, Weiping Liao, Lei Wang, Xun Wu, Xinguang Yu, Jianguo Zhang, Qing Mao, Yuping Wang, Wenbin Li, Xuefeng Wang, Chuanlu Jiang, Xiaoyan Liu, Songtao Qi, Xingzhou Liu, Yan Qu, Jiwen Xu, Weimin Wang, Zhi Song, Jinsong Wu, Zhixiong Liu, Ling Chen, Yuanxiang Lin, Jian Zhou, Xianzeng Liu, Wei Zhang, Shichuo Li, Tao Jiang

**Affiliations:** ^1^ Department of Neurosurgery Chinese PLA General Hospital and PLA Medical College Beijing China; ^2^ Department of Functional Neurosurgery, Beijing Children's Hospital Capital Medical University Beijing China; ^3^ Department of Neuroelectrophysiology Beijing Neurosurgical Institute, Capital Medical University Beijing China; ^4^ Department of Neurosurgery, Beijing Tiantan Hospital Capital Medical University Beijing China; ^5^ Department of Neurosurgery First Affiliated Hospital of PLA General Hospital Beijing China; ^6^ Department of Molecular Neuropathology Beijing Neurosurgery Institute, Capital Medical University Beijing China; ^7^ Department of Neurosurgery, Second Affiliated Hospital Hebei Medical University Shijiazhuang China; ^8^ Department of Neurology, Shanghai Huashan Hospital Fudan University Shaihai China; ^9^ Department of Neurosurgery Tianjin Medical University General Hospital Tianjin China; ^10^ Department of Neurosurgery, Beijing Sanbo Hospital Capital Medical University Beijing China; ^11^ Department of Neurosurgery, Peking Union Medical College Hospital Chinese Academy of Medical Sciences and Peking Union Medical College Beijing China; ^12^ Department of Neurosurgery, Second Affiliated Hospital Army Medical University Chongqing China; ^13^ Department of Neurosurgery First Affiliated Hospital of Nanjing Medical University Nanjing China; ^14^ Department of Neurosurgery, Zhongda Hospital Southeast University Nanjing China; ^15^ Department of Neurosurgery, First Affiliated Hospital Beijing University Beijing China; ^16^ Department of Neurology, Second Affiliated Hospital Guangzhou Medical University Guangzhou China; ^17^ Department of Neurology, First Affiliated Hospital Beijing University Beijing China; ^18^ Department of Neurosurgery, Huaxi Hospital Sichuan University Chengdu China; ^19^ Department of Neurology, Beijing Xuanwu Hospital Capital Medical University Beijing China; ^20^ Department of Neurosurgery, Beijing Shijitan Hospital Capital Medical University Beijing China; ^21^ Department of Neurology, First Affiliated Hospital Chongqing Medical University Chongqing China; ^22^ Department of Neurosurgery Second Affiliated Hospital of Harbin Medical University Harbin China; ^23^ Pediatric Department, First Affiliated Hospital Beijing University Beijing China; ^24^ Department of Neurosurgery, Nanfang Hospital Nanfang Medical University Guangzhou China; ^25^ Epilepsy Center Shanghai Deji Hospital Shanghai China; ^26^ Department of Neurosurgery, Tangdu Hospital Air Force Medical University Xi'an China; ^27^ Department of Functional Neurosurgery, Renji Hospital Shanghai Jiao Tong University Shanghai China; ^28^ Department of Neurosurgery Guangzhou Military General Hospital Guangzhou China; ^29^ Department of Neurology, Xiangya Third Hospital Center South University Changsha China; ^30^ Department of Neurosurgery, Shanghai Huashan Hospital Fudan University Shanghai China; ^31^ Department of Neurosurgery Xiangya Hospital, Center South University Changsha China; ^32^ Department of Neurosurgery The First Affiliated Hospital of Fujian Medical University Fuzhou China; ^33^ Department of Neurology Peking University International Hospital Beijing China; ^34^ China Association Against Epilepsy (CAAE) Beijing China

**Keywords:** adult diffuse glioma, diagnosis and treatment, epilepsy, guideline

## Abstract

**Background:**

Glioma‐related epilepsy (GRE) is defined as symptomatic epileptic seizures secondary to gliomas, it brings both heavy financial and psychosocial burdens to patients with diffuse glioma and significantly decreases their quality of life. To date, there have been no clinical guidelines that provide recommendations for the optimal diagnostic and therapeutic procedures for GRE patients.

**Methods:**

In March 2017, the Joint Task Force for GRE of China Association Against Epilepsy and Society for Neuro‐Oncology of China launched the guideline committee for the diagnosis and treatment of GRE. The guideline committee conducted a comprehensive review of relevant domestic and international literatures that were evaluated and graded based on the Oxford Centre for Evidence‐Based Medicine Levels of Evidence, and then held three consensus meetings to discuss relevant recommendations. The recommendations were eventually given according to those relevant literatures, together with the experiences in the diagnosis and treatment of over 3000 GRE cases from 24 tertiary level hospitals that specialize in clinical research of epilepsy, glioma, and GRE in China.

**Results:**

The manuscript presented the current standard recommendations for the diagnostic and therapeutic procedures of GRE.

**Conclusions:**

The current work will provide a framework and assurance for the diagnosis and treatment strategy of GRE to reduce complications and costs caused by unnecessary treatment. Additionally, it can serve as a reference for all professionals involved in the management of patients with GRE.

## 1 INTRODUCTION

Glioma‐related epilepsy (GRE) is defined as symptomatic epileptic seizures secondary to gliomas. The epileptogenesis of GRE involves various factors including tumor location, tumor histology, microenvironment, and specific genetic alterations.^1^, ^2^, ^3^, ^4^, ^5^ GRE is volatile, unpredictable, closely related to the progression/recurrence of gliomas,^6^ and accordingly places heavy financial and psychosocial burdens on patients and their families.^7^ Moreover, the effect of current conventional treatment strategy for GRE, which consists of antiepileptic drugs (AEDs) and anti‐tumor therapies, is unsatisfactory, despite the above treatments, seizures cannot be effectively controlled in 20%‐40% of patients.^1^, ^8^

Low‐grade gliomas (LGG) are highly epileptogenic and epilepsy is the most common initial symptom occurring in 65%‐90% of cases.^9^, ^10^, ^11^ Isocitrate dehydrogenase 1 (IDH1) mutation, younger age (<38 years), and cortex involvement have been proposed to be associated with a higher frequency of preoperative GRE in LGGs.^9^, ^12^ Over 50% of GRE are drug‐resistant preoperatively, and postoperative seizure freedom rates range from 43 to 87 percent depending on the extent of resection.^13^ In addition to gross‐total resection, older age, generalized seizures, shorter history of seizures and low‐expression of Ki‐67 have been identified as predictors of favorable postoperative seizure control.^14^, ^15^ As for high‐grade gliomas (HGG), the incidence rate of GRE is approximately 40%‐64%.^16^, ^17^ Over 70% of glioblastoma (GBM) patients with GRE preoperatively can become seizure‐free in the early stage after tumor resection, and total resection is still a positive predictor for postoperative seizure control.^16^, ^18^ Additionally, postoperative GRE relapse in HGG is typically correlated with tumor recurrence/progression.^6^ It is also worth noting that no matter in LGG or GBM patients, preoperative GRE is usually correlated with prolonged overall survival.^19^

According to the 2016 World Health Organization (WHO) classification,^20^ the diffuse gliomas include WHO grade II and III astrocytic tumors, grade II and III oligodendrogliomas, and grade IV GBMs. In the current guideline, LGGs are referred to WHO grade II adult diffuse gliomas, while HGGs are referred to WHO grade III‐IV adult diffuse gliomas.

## 2 THE DIAGNOSTIC PROCESS FOR GRE

It is important to recognize that the diagnostic process of GRE should include the diagnoses of both glioma and epilepsy, and the identification of the correlation between them.

The diagnosis of glioma necessitates localization and pathological diagnosis. A patient should receive appropriate history‐taking and physical clinical evaluation at the initial visit. Magnetic resonance imaging (MRI) is essential for preoperative diagnosis, and the conventional scanning sequences should include T1‐weighted, T2‐weighted and contrast‐enhanced T1‐weighted imaging, diffusion‐weighted imaging (DWI), perfusion‐weighted imaging (PWI) and fluid‐attenuated inversion recovery imaging (FLAIR). Moreover, magnetic resonance spectroscopy (MRS), computed tomography (CT), and positron emission tomography (PET) are also helpful supporting methods for the evaluation of glioma. If the tumor involves the eloquent cortex, diffusion tensor imaging and functional MRI should be utilized for the localization of cortical functional areas and fiber tracking.^21^, ^22^ For making a definitive diagnosis of glioma, a surgery/biopsy with subsequent pathological evaluation is necessary. A comprehensive pathological evaluation for glioma should include both histopathological and molecular pathological examinations according to the 2016 WHO classification.^20^ Special attention should be paid to IDH1 mutation status, which is closely associated with GRE, especially for LGG patients.^12^, ^23^

As for the diagnosis of epilepsy, seizure history and clinical signs of epileptic seizures should be conventionally documented for patients with glioma.^2^ Moreover, a 2‐hour video electroencephalogram (EEG), including a non‐rapid eye movement stage I‐II sleep EEG, should be performed for patients with definite or possible epileptic seizures.^2^ Seizure type should be classified according to the 2017 International League Against Epilepsy (ILAE) guidelines.^24^

**Figure 1:**
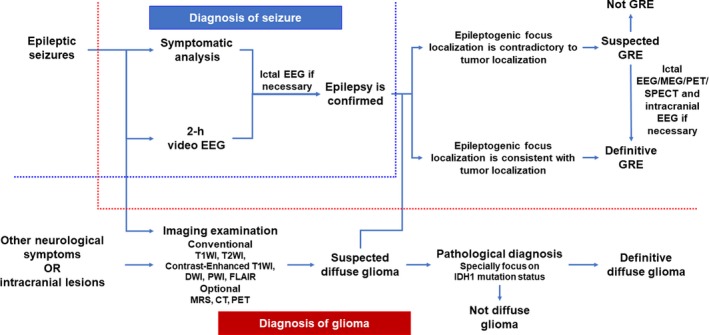
Diagnostic flowchart for glioma‐related epilepsy

Overall, a preoperative diagnosis of GRE can be made based on clinical signs, EEG and imaging findings. Ictal EEG can be used for the differential diagnosis of non‐epileptic attacks in patients without typical clinical seizures or interictal epileptiform discharges; furthermore, it should also be performed when the clinical signs and interictal epileptiform discharges are contradictory to the localization of tumor on MRI.^25^ PET, ictal single‐photon emission CT (SPECT), and magnetoencephalography (MEG) can also be used to localize the epileptogenic zone, if necessary.^26^, ^27^ If various diagnostic reports show no significant correlation between tumor and epilepsy, intracranial EEG can be used to determine the relationship between them.^2^ Figure 1 shows the diagnostic flowchart for GRE.

## 3 TREATMENT OF GRE

### 3.1 Antiepileptic drugs

The administration of AEDs for glioma patients should be initiated as soon as possible after a definite epileptic seizure. The selection of AEDs mainly depends on the seizure type and should follow an individualized treatment plan with adequate drug dosage and duration.

A fundamental principle for AED use in GRE patients is that hepatic enzyme‐inducing AEDs should be avoided for patients receiving chemotherapeutic agents.^28^, ^29^ Among various AEDs, levetiracetam (LEV) and valproic acid (VPA) can be administrated conveniently with various dosage forms to improve seizure control, survival and quality of life for GRE patients, and are accordingly recommended for the monotherapy of GRE patients.^8^, ^28^, ^30^, ^31^ If seizure control cannot be achieved by LEV or VPA alone, polytherapy with LEV and VPA is recommended.^8^, ^32^ It must be noted that as the limitations of available clinical studies, particularly the heterogeneity in terms of dose and duration of drug administration, it is not recommended to use LEV or VPA for reasons other than seizure control in glioma patients.^33^ Additionally, lacosamide has a greater curative effect and fewer side effects in GRE patients resistant or intolerant to other AEDs.^34^

The prophylactic application of AEDs has been a controversial issue for a long time.^1^, ^29^, ^35^, ^36^ For patients without preoperative GRE, the vast majority of clinical evidences supported that the prophylactic application of AEDs during the perioperative period had no benefit.^37^, ^38^ Even for patients with preoperative GRE, there were also plenty of clinical trials suggesting that prophylactic AED use following surgery did not influence the rate of perioperative seizures.^36^ And in 2000, the American Academy of Neurology recommended against prophylactic AED use for patients with brain tumor.^39^ However, according to a survey published in 2017, AED prophylaxis was still routinely used for patients with brain tumor in actual practice.^40^ Such disconnection between the recommendation and clinical practice may be due to the lack of well‐designed contemporary clinical trials.^35^, ^40^ For now, we should be cautious in dealing with this issue; using prophylactic AEDs in certain high‐risk subgroups would be more appropriate. In the current guideline, we recommend that for patients with preoperative GRE, early postoperative AED application is generally acquired; for patients without preoperative GRE, perioperative prophylactic AEDs should be applied in the presence of the following high‐risk factors: (a) frontal or temporal lobe gliomas^41^; (b) chemotherapeutic drug polymer implants during surgery^42^; (c) cortical gliomas or severe cortical injury during tumor resection; (d) gliomas with an oligodendroglial component^43^; (e) recurrent or progressive malignant gliomas; (f) an extended surgical procedure (cortex exposed for over four hours) or anticipated postoperative brain edema/cerebral cortex infarction.

AED withdrawal is also a complex issue for glioma patients. Unlike patients with idiopathic epilepsy, for GRE patients, seizure risk is highly influenced by tumor status and anti‐tumor treatment, and therefore makes predicting the precise seizure risk very difficult. In addition, the side effects of AEDs, the financial, and psychosocial reasons should also be considered. A newly published prospective study suggested that AED withdrawal should only be considered in carefully selected patients with a low risk of tumor progression, even the patients had experienced long‐term seizure freedom.^44^ As for the timing of AED withdrawal, we recommend that for patients without preoperative and postoperative seizures, withdrawal of prophylactic AEDs is feasible at 2 weeks after surgery. Patients without preoperative GRE can gradually withdraw AEDs after a 3‐month administration period in those with a single early‐stage postoperative seizure, however, for patients with repeated postoperative seizures, AED withdrawal should be delayed after a minimum seizure freedom period of 1 year. For patients with preoperative GRE, patients can withdraw AEDs after a minimum of 1 year of seizure freedom when their seizure histories are shorter than 6 months and tumors are completely removed, however, for those with a long seizure history, incomplete tumor resection, distant epileptiform EEG discharges, preoperative drug‐resistant seizures or focal seizures without a loss of consciousness, we recommend that the optimal timing of AED withdrawal should be at least 2 years without seizures after the surgery and needs to be considered carefully.^44^, ^45^ AED withdrawal is not recommended for two subgroups in any case: (a) all GBM patients^46^; (b) other HGG patients (patients with anaplastic glioma) with incomplete tumor resection or intractable postoperative seizures.

### 3.2 Surgery

It is important to be clear that up to now, the primary purpose of surgery in patients with glioma has been oncologic tumor control but not seizure control. However, neurosurgeons have realized the importance of seizure control for patients with glioma and regarded it as a second goal of surgery. In terms of seizure control, gross‐total resection is better than sub‐total resection.^9^, ^13^, ^15^, ^47^ A recent study showed that for LGG patients with preoperative epilepsy, the postoperative seizure control was more likely when the extent of resection was over 91%.^48^ Additionally, “supratotal” resection can achieve better seizure control than even gross‐total resection.^49^ Accordingly, for patients with GRE, the maximal safe resection is helpful to improve not only local tumor control and survival but also postoperative seizure control. For patients with tumors involving the eloquent cortex, gross‐total resection is not feasible, the most advanced technologies should be used to achieve removing the tumor maximally to reduce postoperative seizures while protecting brain function, for instance, “engraving surgery” can be effective. Additionally, intraoperative electrocorticography is recommended for LGG patients with preoperative refractory GRE to guide the resection of the epileptogenic area and to improve the postoperative seizure outcome.^50^

Epilepsy relapse or aggravation could be related to tumor recurrence or progression in patients with GRE.^16^ Postoperative MRI (combined with contrast‐enhancement) should be performed within 24‐72 hours after surgery to evaluate the extent of resection, as this influences the incidence and timepoint of postoperative seizures greatly. Relapse of epilepsy following a long‐term postoperative seizure‐free period may suggest tumor recurrence.^6^ If tumor recurrence is accompanied by frequent drug‐resistant seizures, an operation is feasible after a comprehensive assessment of the patient's condition. If the patient suffers from postoperative seizures without evidence of tumor recurrence, an evaluation following the principle of refractory epilepsy should be performed. In cases of drug‐resistant GRE, surgery should be considered when the quality of life of patient is significantly decreased due to frequent seizures.^2^

### 3.3 Management of intraoperative and early postoperative epilepsy

Direct electrical stimulation for functional cortical or subcortical mapping may lead to epileptic seizures during awake craniotomy of gliomas, intraoperative seizures are usually partial seizures and the incidence is approximately 3.2%‐15.5%.^51^, ^52^, ^53^ Patients with the following risk factors are more likely to experience intraoperative seizures: younger age, frontal lobe (mainly supplementary motor area) involvement, preoperative seizure history, treatment with multiple AEDs preoperatively, and IDH1 mutation.^51^, ^54^, ^55^ Intravenous injection of LEV or VPA can be prophylactically used for these high‐risk patients. Once intraoperative seizures occur, the surgeons should stop the stimulation and irrigate the cortex with ice‐cold Ringer's solution or saline immediately.^56^ In general, the intraoperative seizures can be quickly resolved by the above procedure, in case of seizure persistence, benzodiazepines should be injected in a timely manner to stop the seizure. Additionally, intraoperative electromyographic monitoring could be used for early detection of potential seizure onset.

In the case of immediate or early postoperative seizures, electrocardiography and routine blood, urinalysis, blood glucose, hepatic and renal function, and electrolyte tests should be performed to exclude non‐epileptic attacks caused by cardiac incident, hypoglycemia, or electrolyte disturbance. Subsequently, CT or MRI should be performed to exclude intracranial hemorrhage and infarction after the initial postoperative seizure is controlled. If the patient's condition allows, 2‐hour EEG monitoring should be used to observe the correlation between abnormal epileptiform discharges and brain edema/residual tumor. There is no need to change the therapeutic strategy for patients receiving AEDs, while monotherapeutic AEDs should be applied to those who do not receive prophylactic AEDs perioperatively.^16^ The blood concentration of AEDs should be monitored when multiple episodes are observed and add‐on treatment with another type of AED must be considered when seizures are poorly controlled.

**Figure 2:**
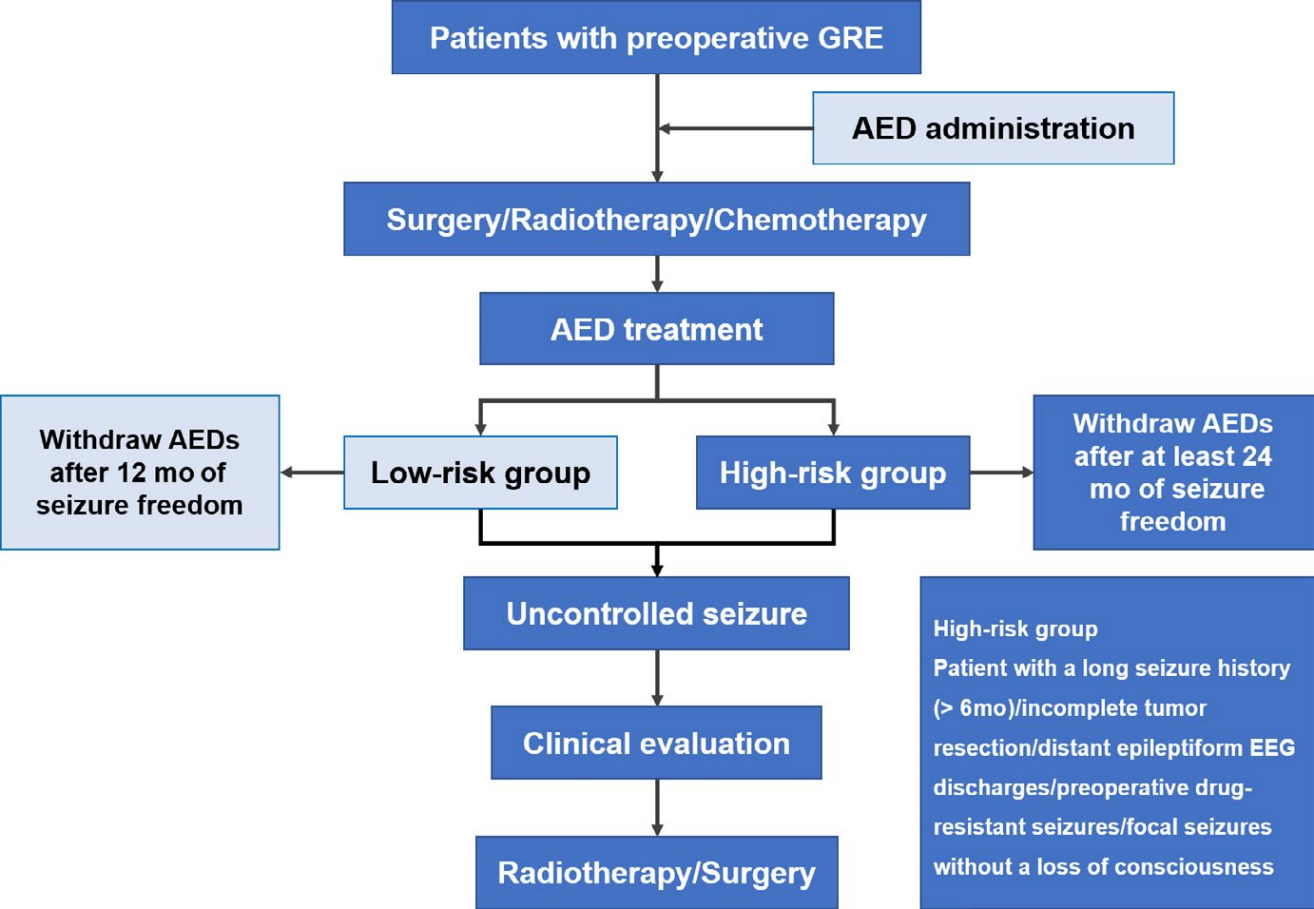
Process flowchart for patients with preoperative glioma‐related epilepsy. For glioblastoma patients and other high‐grade gliomas patients with incomplete tumor resection or intractable postoperative seizures, antiepileptic drug withdrawal is not recommended

**Figure 3:**
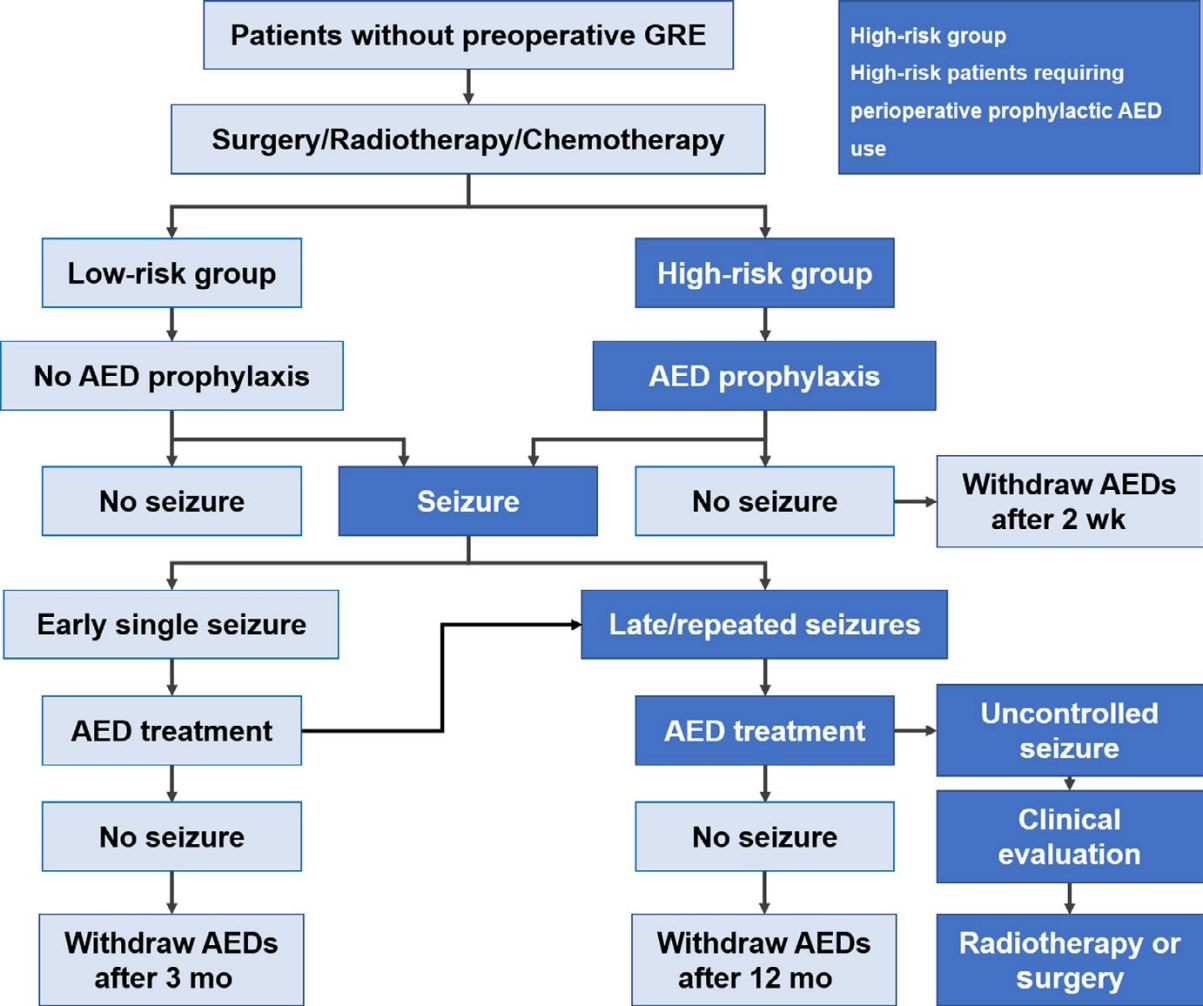
Process flowchart for patients without preoperative glioma‐related epilepsy. For glioblastoma patients and other high‐grade gliomas patients with incomplete tumor resection or intractable postoperative seizures, antiepileptic drug withdrawal is not recommended. Early seizures, seizures appear within 2 wks after surgery; Late seizures, seizures appear over 2 wks after surgery

Figures 2 and 3 show the process flowcharts for patients with preoperative GRE and those without, respectively.

### 3.4 Radiotherapy

All relevant clinical evidences showed that radiotherapy had a significant effect on inhibiting GRE.^57^ For patients with GRE, the radiotherapy strategy is the same as those without GRE and postoperative radiotherapy at the early stage is recommended.^58^, ^59^ It is noteworthy that seizure control is more often achieved in patients with a long seizure duration before the start of radiotherapy and is not strictly associated with tumor shrinkage on MRI.^60^ Additionally, glioma patients with frequent refractory epilepsy and intolerance to surgery can receive radiotherapy, irrespective of tumor relapse.

### 3.5 Chemotherapy and other treatments

Similar to radiotherapy, chemotherapy (procarbazine‐lomustine‐vincristine or temozolomide) is also correlated with improved seizure control in 30%‐100% of patients with GRE, regardless of surgical resection.^57^

Moreover, an adjuvant ketogenic diet may be useful to suppress glioma cell proliferation, as well as reduce seizure frequency and severity.

### 3.6 Note

Relevant recommendations are summarized in Table 1. The levels of evidence and grades of recommendation were evaluated and given according to the Oxford Centre for Evidence‐based Medicine Levels of Evidence and Grades of Recommendation (https://www.cebm.net/2009/06/oxford-centre-evidence-based-medicine-levels-evidence-march-2009/). For some controversial issues, we also proposed Chinese expert consensus as references for clinical practice.

**Table 1:** Conclusion and recommendations

Recommendations	Level of evidences	Grade of recommendation
Diagnosis		
MRI is essential to obtain a definite preoperative diagnosis of glioma	Ⅱb	B
Pathological evaluation for glioma should be performed according to 2016 WHO classification	Ⅰa	A
Special attention should be paid to the IDH1 mutation status	Ⅱa	B
Seizure type should be classified according to the 2017 ILAE guidelines	Ⅰa	A
AEDs		
The administration of AEDs should be initiated as soon as possible after a definite seizure	Expert consensus	For reference
Hepatic enzyme‐inducing AEDs should be avoided for patients undergoing chemotherapy	Ⅰb	A
LEV and VPA are recommended for the monotherapy of GRE patients	Ⅰb	A
Polytherapy with VPA and LEV can be more effective when monotherapy is unsatisfactory	Ⅱb	B
For patients with preoperative GRE, early postoperative AED application is generally acquired	Expert consensus	For reference
For patients without preoperative GRE, prophylactic AEDs is acquired for high‐risk subgroups	Expert consensus	For reference
The timing of AED withdrawal should be carefully considered (see 2.1, paragraph 4)	Expert consensus	For reference
Surgery and management of intraoperative and early postoperative epilepsy		
Maximal safe resection is helpful to improve postoperative seizure control	Ⅱa	B
Intraoperative electrocorticography is recommended for LGG patients with refractory GRE	Ⅱb	B
Irrigating the cortex with ice‐cold Ringer's solution or saline is useful to control intraoperative seizures	Ⅳ	C
Radiotherapy, chemotherapy, and other treatments		
Radiotherapy has a significant effect on inhibiting GRE	Ⅱa	B
Chemotherapy is also effective for the control of GRE	Ⅱa	B

AEDs, antiepileptic drugs; GRE, glioma‐related epilepsy; ILAE, International league against epilepsy; LEV, levetiracetam; LGG, low‐grade gliomas; MRI, magnetic resonance imaging; VPA, valproic acid.
